# ChromaCorrect: prescription correction in virtual reality headsets through perceptual guidance

**DOI:** 10.1364/BOE.485776

**Published:** 2023-04-21

**Authors:** Ahmet H. Güzel, Jeanne Beyazian, Praneeth Chakravarthula, Kaan Akșit

**Affiliations:** 1 University of Leeds, School of Computing, Leeds, UK; 2 University College London, Computer Science Department, London, UK; 3 Princeton University, Princeton, USA

## Abstract

A large portion of today’s world population suffers from vision impairments and wears prescription eyeglasses. However, prescription glasses cause additional bulk and discomfort when used with virtual reality (VR) headsets, negatively impacting the viewer’s visual experience. In this work, we remedy the usage of prescription eyeglasses with screens by shifting the optical complexity into the software. Our proposal is a prescription-aware rendering approach for providing sharper and more immersive imagery for screens, including VR headsets. To this end, we develop a differentiable display and visual perception model encapsulating the human visual system’s display-specific parameters, color, visual acuity, and user-specific refractive errors. Using this differentiable visual perception model, we optimize the rendered imagery in the display using gradient-descent solvers. This way, we provide prescription glasses-free sharper images for a person with vision impairments. We evaluate our approach and show significant quality and contrast improvements for users with vision impairments.

## 1. Introduction

Virtual Reality (VR) headsets are becoming increasingly popular amongst consumers, encouraging researchers to conceptualize and build technologies enabling fully immersive remote experiences [1]. However, most recent developments overlook the prevalence of refractive vision problems such as myopia, hyperopia, or astigmatism are among potential VR users at least 23.9%, 8.4%, and 33% of the population, respectively [2]. Moreover, while the current near-eye display research is focused on the miniaturization of the headset to eyeglasses form-factor [3,4], wearing prescription glasses under a VR headset causes uncomfortable viewing experiences that break the feeling of immersion.

**Fig. 1.**
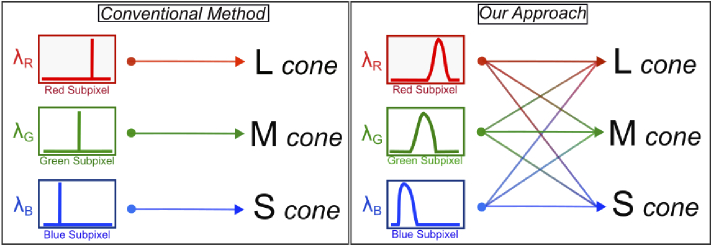
A comparison of the differences between our approach and conventional methods through a simple visualization

The majority of hardware-based methods for prescription correction [5–7] could result in VR/AR headsets that are bulkier and more expensive, requiring the upgrading of components with new devices. On the other hand, algorithmic approaches to prescription correction enable tackling the prescription issue without the need for specialized components and with the benefit of software updates [8]. While we acknowledge that hardware solutions provide high-quality prescription correction solutions, we argue that algorithmic approaches may offer convenient, programmable, and practical alternatives without complex hardware. Specifically, using less hardware in algorithmic approaches could help mass adoption of VR/AR technology [8–13]. In the conventional algorithmic approach, it is assumed that each color channels of RGB correspond one-to-one relation with retinal cells (see Fig. 1). In this situation, it may be appropriate to view the backlight spectrum of the targeted display as a coherent light source. However, this may not be the case, as backlight spectrums typically have broadband intensity values across different wavelengths, which are processed by retinal cells with broadband responsivity spectra.

**Fig. 2.**
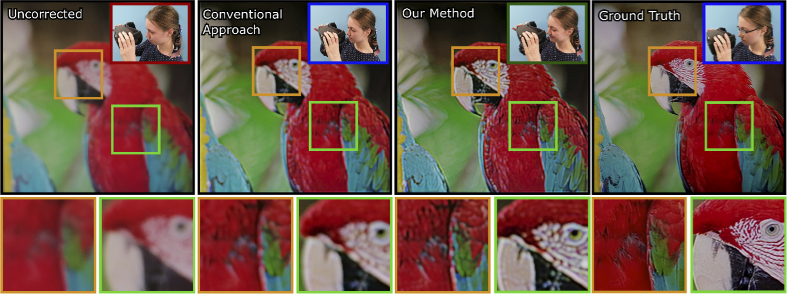
Perceptual Guidance in Prescription Correction. We provide a differentiable perception model for optimizing images that compensate for user prescription and improve the perceived contrast and color in images. Here, we show images as captured by a camera on a reference display where the images are intentionally defocused to mimic an eye with common refractive errors. Without any prescription correction, the perceived images appear blurry due to defocus caused by refractive errors (first column). The second column captures the performance of the conventional algorithmic approach to prescription correction [8] for the same refractive error. Our proposed computational approach to algorithmic prescription compensation improves the perceived images, both in color and contrast, as can be seen in the third column. For reference, we provide a ground truth photograph focused at the display plane as in the fourth column, resembling what a user would see with their prescription lenses incorporated into the virtual reality headset. Source image is from Rich Franzen [14].

We propose replacing the conventional RGB image-based pipeline with a CIE LMS color space-enhanced pipeline to improve contrast and color (see Fig. 1). Our work offers a new perceptually-guided algorithmic approach to prescription correction while eliminating the need for corrective lenses. To this end, we first study the low-level workings of the Human Visual System, i.e., how different types of cone cells respond to various wavelengths of light. We then model the display’s specific light spectrum (e.g. subpixels emitting various wavelengths) and the associated response of cone cells on the retina. Hence, we build an end-to-end differentiable perception model that helps us to simulate how a user with a Point-Spread Function (PSF) model with Zernike polynomials [15] perceives images on a specific display. Finally, our end-to-end perception framework optimizes display rendering to produce an in-focus image for a user with vision impairments (see Fig. 2). Specifically, our work makes the following contributions:

• **Perceptually guided Prescription Correction.** We incorporate the display specific color perception and PSF of a user into a new differentiable model to ensure that the optimized image’s contrast and color characteristics are distinctly enhanced in visual perception.
• **Learned Prescription Correction.** We train a Convolutional Neural Network (CNN) to estimate optimal images for prescription correction, enabling prescription correction at interactive rates.
• **Evaluation on Actual Displays.** We analyze our findings on actual display hardware and demonstrate real-life use cases.

## 2. Related work

Researchers have previously attempted to compensate for refractive vision problems for glasses-free experience in displays. We summarize most relevant papers here in Table 1.

**Table 1.** Comparison of prescription correction techniques. Many of the solutions for prescription correction either fail to provide good image quality or require bulky hardware components affecting user comfort negatively. We take an algorithmic approach utilizing an accurate perception model of the human visual system, leading to improved image quality and real-time image generation. SW refers to Software while HW refers to Hardware in this table.

Name	Method	Perceptual Guidance	Realtime	Image Quality	Display Type
Multi-domain [9]	SW	Preliminary	No	Poor	Desktop
Constrained Total Variation^*a*^ [8]	SW	Preliminary	No	Poor	Desktop
Tone Mapping [10]	SW	Preliminary	No	Poor	Desktop
Network [11]	SW	No	No	Poor	Desktop
Vision Enhancement [12]	SW	No	No	Poor	AR
SharpView [16]	SW	No	No	Poor	AR
FocusAR [7]	HW	No	Yes	Good	AR
Autofocals [17]	HW	No	Yes	Good	AR
Phase Modulated [18]	HW	No	Yes	Good	AR
RectifEye [19]	HW	No	Yes	Good	VR
Alvarez Lenses [20]	HW	No	Yes	Good	VR
Software [13]	SW	No	Yes	Poor	VR
Ours	SW	Color Vision	Yes	Fair	VR

^*a*^ This technique is referred to as the conventional method throughout the paper.

### Programmable Prescription Lenses.

Utilizing focus-tunable lenses that may be adjusted to the user’s prescription is a common technique [21], especially in displays such as VR headsets where the users view a display through magnifying lenses [7,17,19,20]. An alternative to these approaches, phase-only spatial light modulators, could also be used to form a programmable prescription correction lens [18]. Beyond requiring customized hardware, these techniques would also require eye-tracking and depth sensor data of a scene to operate, leading to more demands in hardware.

### Computational Displays.

Altering the display hardware and image acquisition technologies could help with prescription correction [22]. Huang, Lanman, Barsky, Raskar, et al. [23] address extreme contrast loss and ringing artifacts in algorithmic correction techniques by utilizing a stack of semi-transparent, light-emitting layers for LCDs. Wu and Kim [5] embed free-form image combiners inside prescription lenses to create customizable Augmented Reality (AR) displays. Pamplona, Oliveira, Aliaga, Raskar, et al. [24] implements 4D light field displays to move the solution to a higher-dimensional (light field) space, where the inverse problem is well-posed. To overcome this limitation in resolutions in Pamplona’s work [24], Huang, Lanman, Barsky, Raskar, et al. [25] propose a 4D prefiltering algorithm that can provide higher contrasts and resolutions. The described approach [24] has a significant drawback, namely that the PSF of an eye with refractive errors is typically a low-pass filter and, as such, irrevocably cancels higher frequencies from the original image. Moreover, holographic vision correction [26,27] is superior to conventional approaches, including light field displays. Curious readers could consult to survey by Aydinoğlu, Kavakli, Sahin, Artal, Ürey, et al. [28] for more on these holographic displays.

### Algorithmic Prescription Correction.

Refractive vision impairments of the eye are commonly modeled by constructing a PSF that represents how the eye as an optical system transmits a point on the object to a point on the retina. The spatially varying PSF is convolved with the image of the object to produce the image formed on the retina. Performing the inverse operation, i.e., deconvolving the image with the retinal PSF, could help produce an image that forms clearly on the retina when observed. Alonso, Barreto, Jacko, et al. [29] verifies the possibility of such an image correction technique by constructing a simple artificial eye and comparing the image it forms when viewing a standard and a corrected image. They also propose an ad-hoc solution to mitigate contrast loss and “ripples” or ringing artifacts [9]. Montalto, Garcia-Dorado, Aliaga, Oliveira, Meng, et al. [8] present constrained total variation to decrease ringing artifacts in the corrected image while sharpening the image’s edges, thereby producing an image with high contrast along sharp edges. Ye, Ji, Zhou, Kang, J. Yu, et al. [10] focus on finding a ringing-free image with higher contrast in locations important to Human Visual System, while tolerating more blurriness elsewhere. Tanaka, Kawano, et al. [11] uses a CNN-based pipeline for prescription correction along with Zernike-based visual aberration modeling. Li, Suo, Zhang, Yuan, Dai, et al. [30] feed an aberrated image and a map of a PSF for multiple subregions, to account for spatially variant aberrations into a deep neural network and train it for image correction on a variety of lenses. Similar image correction techniques have been applied to VR headsets. Itoh, Klinker, et al. [12] corrects the defocus aberration for optical see-through headsets by overlaying a compensated image in the user’s view. Xu, Li, et al. [13] use gradient-based priors to achieve real-time visual aberration correction for VR HMDs. Oshima, Moser, Rompapas, Swan, Ikeda, Yamamoto, et al. [16] describe real-time defocus correction for optical see-through HMDs, which is caused by focal rivalry: the simultaneous viewing of real and virtual content.

Perceptual considerations in displays and graphics systems are becoming commonplace in relevant research branches. The surveyed research work does not provide a complete model of Human Visual System in their solutions, leading to either poor image quality or demanding hardware. We believe our work resembles the first attempt to enhance algorithmic solutions in the literature by bridging the gap between perceptual modeling by means of color vision and prescription correction.

## 3. Perceptually guided prescription correction

**Fig. 3.**
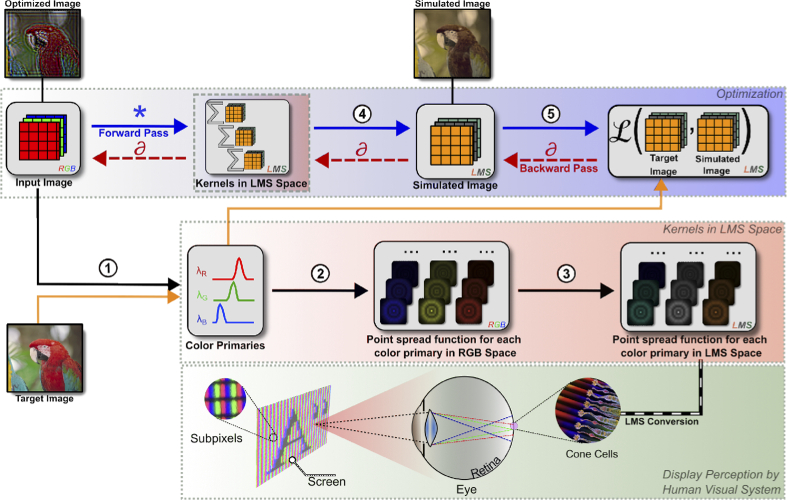
Prescription correction using a perceptually guided computational model and a differentiable optimization pipeline. (1) A screen with color primaries (RGB) displays an input image. (2) A viewer’s eye images the displayed image onto the retina with a unique Point Spread Function (PSF) describing the optical aberrations of that person’s eye. (3) Retinal cells convert the aberrated RGB image to a trichromat sensation, also known as Long-Medium-Short (LMS) cone perception [31]. (4) Our optimization pipeline relies on the perceptually guided model described in previous steps (1-3). Thus, the optimization pipeline converts a given RGB image to LMS space at each optimization step while accounting for the PSFs of a viewer modelled using Zernike polynomials. (5) Our loss function penalizes the simulated image derived from the perceptually guided model against a target image in LMS space. Finally, our differentiable optimization pipeline identifies proper input RGB images using a Stochastic Gradient Descent solver [32].

We introduce a differentiable framework for modeling the display and human visual perception, encapsulating display-specific parameters, color and visual acuity of human visual system and the user-specific refractive errors. State-of-the-art methods use prescription correction by reconstructing the precorrected images by using RGB channel images convolved with pre-calculcated PSF. Our framework allows for optimizing prescription compensated rendered imagery on standard displays using a gradient-based policy with novel display-specific perceptually guided loss functions (Section 3.1). We rely on Zernike polynomials (Section 3.2) for describing user-specific retinal point spread functions [27] within the forward model to represent optical aberrations in the Human Visual System (Section 3.3). On overview of our entire display-visual perception model and the optimization process is depicted in Fig. 3.

### 3.1. Modeling display-specific visual perception

We characterize our target display and device a computational model to transform the displayed imagery on the target display into imagery as perceived by the Human Visual System.

#### Characterizing target display.

A given display has three types of emission spectra, $\lambda_{R},\lambda_{G},\lambda_{B}$ , for their red, green, and blue channel pixels, respectively. We converted the measured spectrum of the targeted display into two-dimensional arrays, with one dimension representing wavelength and the other representing the normalized spectrum for each color primary. We use a multi-layer perceptron network that act as general function approximator for fitting a robust representation of raw data. Implementation of this can be found in (See odak.learn.tools.multi_layer_perceptron() in [33,34]). Once we produce 2-D array-based data for color primaries, we utilize it to investigate the color perception responses of the Human Visual System.

#### Converting color primaries to perceived colors.

Human retinal cells can be broadly classified into rods and cones. Cone cells, which are primarily responsible for color perception in the Human Visual System, are of three different subtypes: Short (S), Medium (M), and Long (L) cells. Each of them differs in its sensitivity to different wavelengths of light. The L, M, and S cones reduce wavelengths of incoming light into trichromat values by integrating them over their response functions [35]. Note that perception in Human Visual System is contrary to modeling camera-display response where red, green, and blue wavelengths are independently measured on the camera sensor or the human retina. The following steps show how to convert an input color image displayed on a target display to the corresponding cone response:

(1) $$\left[\begin{array}{c} I_{L}\\ I_{M}\\ I_{S} \end{array}\right]=[\begin{array}{ccc} L_{R} & L_{G} & L_{B}\\ M_{R} & M_{G} & M_{B}\\ S_{R} & S_{G} & S_{B} \end{array}]\;\left[\begin{array}{c} I_{R}\\ I_{G}\\ I_{B} \end{array}\right]\;,$$

where $I_{R}$ , $I_{G}$ , $I_{B}$ represents red, green and blue pixel values of an input image, and $I_{L}$ , $I_{M}$ , $I_{S}$ represents L, M and S cone activation values for each pixel of the displayed image. From the generalized formula above, we provide a sample conversion for $L_{R}$ as in the following equation,

(2) $$\sum_{\lambda_{R}}\;\lambda_{L}\lambda_{R}=L_{R},$$

where $\lambda_{L}$ represents L cone sensitivity function, $\lambda_{R}$ represents red pixel emission spectrum function for a targeted display, and $L_{R}$ represents L cone output for the displayed red pixel. Similarly, L cone sensitivity functions for green and blue pixel emissions can be computed. Thus, L, M and S cone sensitivity functions can be computed for the three different subpixel emissions. After computing the cone sensitivity functions, we apply the conversion from the color opponency model proposed by Schmidt, Neitz, et al. [36] to represent a complete perception model,

(3) $$\left[\begin{array}{c} I_{(M+S)-L}\\ I_{(L+S)-M}\\ I_{\bar{\left(L+M+S\right)}} \end{array}\right]=\left[\begin{array}{c} (I_{M}+I_{S})-I_{L}\\ (I_{L}+I_{S})-I_{M}\\ \bar{(I_{L},I_{M},I_{S}}) \end{array}\right]\;,$$

where $I_{(M+S)-L}$ , $I_{(L+S)-M}$ , $I_{\;\bar{\left(L,M,S\right)}}$ represents the three channels of the image sensed in the color-opponency space.

### 3.2. Computing point spread functions from color primaries

The point spread function for the HVS with visual aberrations can be defined over several wavelengths of light. Therefore, we can sample a set of wavelengths from each color primary, calculate PSFs for each and use a weighted sum of the PSFs to obtain a single, combined PSF for each color primary,

(4) $$\text{PSF}(x,y,c)=\sum_{c}\sum_{\lambda_{c}}w_{\lambda_{c_{i}}}\text{PSF}(x,y,\lambda_{c_{i}})$$

where c represents a particular color primary, PSF(x,y,c) is the PSF for a particular color primary, $\text{PSF}(x,y,\lambda_{c_{i}})$ the PSF for a sampled wavelength in the color primary and $w_{\lambda_{c_{i}}}$ is the weight for that sampled wavelength. The above PSF kernel can be utilized in RGB, or color opponency spaces, depending on designers choices. In our method, we introduce color opponency based PSF formulation (perceptually guided) to improve the perceptual characteristics (contrast, quality) of the retinal image. Equation (4) is extended to formulate LMS based kernel,

(5) $${\text{PSF}}_{\text{lms}}(x,y,\lambda_{c_{i}})=A*\text{PSF}(x,y,\lambda_{c_{i}})$$

(6) $${\text{PSF}}_{\text{lms}}(x,y,c)=\sum_{c}\sum_{\lambda_{c}}w_{\lambda_{c_{i}}}{\text{PSF}}_{\text{lms}}(x,y,\lambda_{c_{i}})$$

where A is the conversion matrix defined in Eq. (1), ${\text{PSF}}_{\text{lms}}(x,y,c)$ is the PSF for a particular color primary with LMS components. Similarly, we modelled the digital camera color primary decoding by using measurements from the display and captured images from the digital camera. In this way, we are able to use digital camera captured images to represent our work in this paper. In the Eq. (5) and Eq. (6), ${\text{PSF}}_{\text{lms}}$ is represented for both the HVS and digital camera RGB decoding. We can now compute the retinal image r(x,y,c) in the LMS space, by convolving ${\text{PSF}}_{\text{lms}}$ with the input image s(x,y,c) ,

(7) $$r(x,y,c)={\text{PSF}}_{\text{lms}}(x,y,c)*s(x,y,c).$$

### 3.3. Optimizing images for prescription correction

In the final step, we aim to optimize an image that passes through the eye’s optical system (modeled as a convolution in Eq. (7)). The eye’s optical system serves as a computational model, utilizing a combination of Zernike polynomials and cone cell responses to the color spectrum perceived by the human eye, defined as HVS modeling. This optimization is done by solving the optimization problem,

(8) $$s\prime \leftarrow \underset{s\notin \emptyset }{\text{arg}\;\min}\;L(\text{PSF}*s,t)$$

where t is the the ground truth image and s′ is the input image optimized for a user’s eye, PSF is a kernel defined in Eq. (4). In our method, we reformulate Eq. (8) to incorporate color opponency space optimization,

(9) $$s\prime \leftarrow \underset{s\notin \emptyset }{\text{arg}\;\min}\;L({\text{PSF}}_{\text{lms}}*s,t_{\text{lms}})$$

where $t_{\text{lms}}$ is the the ground truth image in LMS space and s′ is the input image optimized for a user’s eye, ${\text{PSF}}_{\text{lms}}$ is kernel defined in Eq. (6). To perform the above optimization, we compare images using a loss function (e.g. least-squared error) to calculate the error between the ground truth image and the retinal image, L(r(x,y,c),t(x,y,c)) , where x and y represent image coordinates and c the color channels, which could be in RGB or LMS color opponency spaces. Note that we have also built a learned equivalent of our approach, which we will detail in the Sec. 4.

## 4. Implementation

Our approach is composed of three primary elements: a color perception model, a prescription correction optimization pipeline and a learned model that demonstrates that our differentiable pipeline can be learned. All of these components are implemented on PyTorch [32].

**Table:**
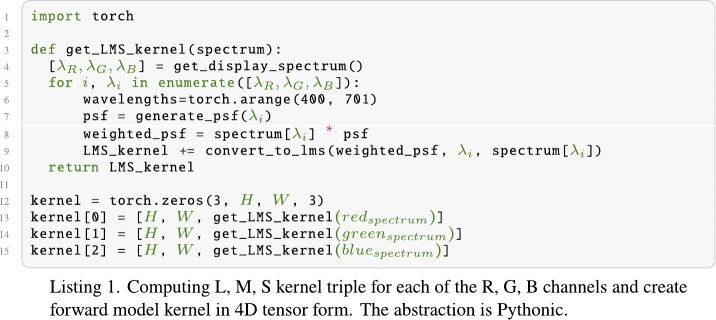


### 4.1. Color perception model

Firstly, we identify the emitted wavelengths from the subpixels of a target display device. For that purpose, we acquire the spectrometer data for a target display consisting of discrete wavelengths and their corresponding intensity values normalized between zero and one. We use Multilayer Perceptron (MLP) to fit a curve on this discrete data to achieve a vector representation of our intensity profile of color primaries with respect to wavelength. Our MLP has 64 hidden layers and converges over 1000 iterations in training with a learning rate of 0.0005. Once we have numerically identified the normalized intensity of each color primaries as a function of wavelength, we use these 2D (intensity, wavelength) vectors to create our color perception based kernel in LMS space. For each color primary, we create the set of PSF based on our Zernike polynomial generator by sampling wavelengths from 400 to 700 with 1 nm intervals. During each sampling step, we create weighted kernels by multiplying the created PSF with the intensity value based on corresponding wavelength from our created 2D vectors for each color primary. After creating the weighted kernel in each sampling step, we obtain LMS cone responses of weighted kernel using the same intensity and wavelength data. To compute LMS cone responses, we use the method explained in section 3.1. In the last step, the set of weighted kernels are summed up to create our color perception based kernel for each color primary forming a 4D tensor as *[Color Primary, H, W, LMS Response]*. Our method differs from the conventional method both in terms of kernel type, and convolution operation.

In conventional method, kernel is a 3D tensor with RGB channels while in our method we use 4D tensor. In this 4D tensor formed kernel, each color primary has LMS triple separately as *[3, H, W, 3]*. The LMS based kernel convolves the image’s each color channel with corresponding each display spectrum LMS responses. This operation computationally more expensive compared to conventional method, since more matrix operation is needed. We provide a pseudo-code for constructing our LMS based kernel as in Listings 1.

### 4.2. Optimization pipeline

We implement a prescription correction optimization pipeline using a modern machine learning library with automatic differentiation [32]. Source code of our implementation is publicly available at https://github.com/complight/ChromaCorrect

*Optimization loop:* The differentiable input RGB image initialized from our target RGB image, and it is passed through the forward model during optimization loop. In forward model, each color channel of initialized input RGB image convolved with the LMS kernel created in computational color pipeline. For example, red channel of input RGB image is convolved with L, M, S channel of red spectrum kernel in LMS space. Other color channels of input RGB image are convolved with the same method. The resulting simulated image represents the image formed on the retina from L, M, S cone activations. The target image is converted to LMS space to calculate L2 loss against the simulated image in LMS space, which is back-propagated through the optimization model to the input RGB image. Our results are obtained using Stochastic Gradient Descent with ADAM [37] as the optimizer. Our method enables the reconstruction of images tailored to an individual’s specific visual needs by allowing the input of eye prescription values for myopia, hyperopia, and astigmatism. The proposed pipeline is available to be used in NVIDIA GPU accelerated computer.

### 4.3. Learned model

We implement a semi-supervised deep learning model capable of reconstructing optimized images from their original RGB versions. We use a U-Net architecture [38] for this purpose. Such a solution is more suitable than an iterative process for achieving real-time applications. But it trades the image quality for a faster rendering speed. Our model comprises two outer layers linked to 8 hidden convolutional layers symmetrically connected by skip connections. Each layer on the contractive path of the model are formed by a double convolution and a max pooling operation. On the expanding path, an up-sampling operation with bilinear interpolation initiates each convolution. During training, batch normalization and ReLU activation are used.

Our model was evaluated on a machine with an NVIDIA GeForce RTX 2070 GPU. The training dataset consists of 20 images of dimension 512 x 512 pixels, the RGB images were obtained from Zhang, Wu, Buades, Li, et al.’s color image processing dataset [39] and the target optimized images were generated using our iterative method. A learning rate of $1\times 10^{-4}$ was used for the training phase, and a conventional mean-squared-error loss function guides the stochastic gradient descent optimization. With convolutional kernels of size 3x3, each input image sees its channels expand from 3 to 92 and up to 1472 at the latent space. The results in Fig. 8 show the comparison of the corrected image between our original pipeline and the neural network’s prediction after over 800 epochs of training. The learned model significantly reduces image generation time, with an average of 2.9 milliseconds per corrected image compared to the original method’s 8.127 seconds, a speed increase of approximately 2800 times. The primary focus of our training with a small dataset is to demonstrate that the network can effectively learn pre-corrected image features, resulting in significantly faster rendering speeds than the proposed optimization-based solution without requiring much training data. However, learned methods could be further investigated with larger datasets in the future.

## 5. Evaluation

**Fig. 4.**
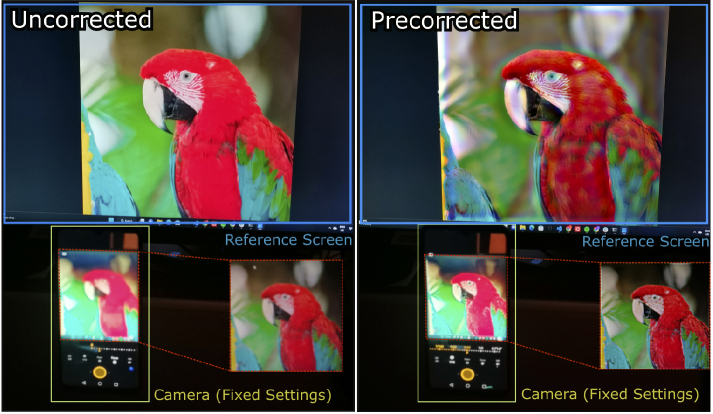
Experimental setup for camera defocus experiments. For every experimental image capture, we fixed the pose, ISO, and focus setting of the camera to ensure a consistent view with a nearsighted prescription of -1.50.

We divided our experiments into two sections. In the first part, we use real hardware to test our methods for defocus prescription. Precorrected images are displayed on a computer monitor as a reference, and a focus-controlled digital camera is used to replicate the view of a myopic eye. In our experiments, we use fixed pose, focus camera to capture images to demonstrate the method’s performance. Upon inputting the desired eye prescription type and value, we generated a pair of reconstructed images using both conventional (RGB) and our proposed (LMS) method. We then captured images from the reference display using a fixed position and defocused camera to assess the superiority of our method over the conventional approach. Figure 4 shows our experimental setup for defocus experiments. Figure 6 shows two different images with the same nearsighted prescription -1.50.

**Fig. 5.**
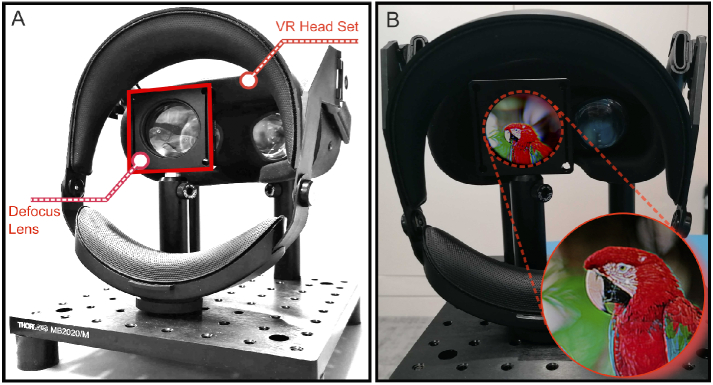
Testbed used in our evaluations. (A) We use a virtual reality headset and a camera to capture images from our virtual reality headset. To emulate a prescription problem in the visual system, we use a defocus lens. (B) We take pictures with fixed pose and camera focus from behind the defocus lens to evaluate reconstructed images.

Experiments show that we improved contrast and color compared to conventional method. In fact, our method is not able to produce same quailty with the target image. We also used Oculus Quest 1 virtual reality headset, and we placed a defocus lens to create artificial prescription for a subjective evidence of our methods performance against conventional method. In our experiments, we utilized an eye relief of 20 millimeters, a camera focus of 250 millimeters (0.25 Diopters), an aperture size of f/1.8 with 13mm pupil size, and a targeted refractive error of -1.5 D to render the captured images and showcase the method’s performance. Our setup is shown in Fig. 5.

**Fig. 6.**
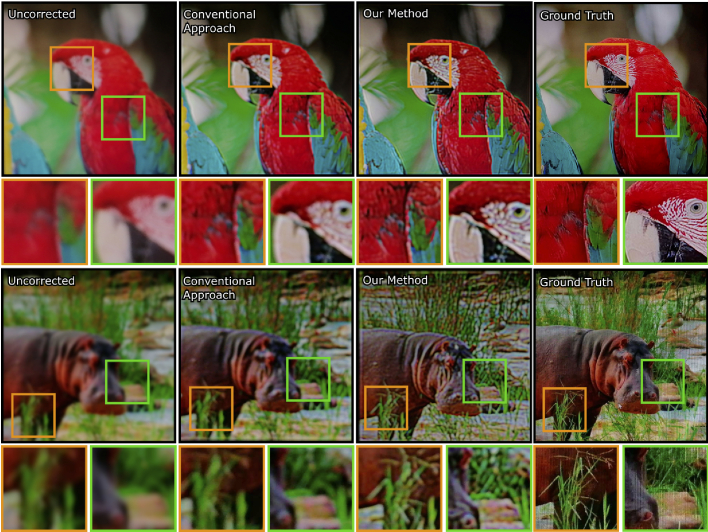
In the absence of prescription correction, images appear blurry as a result of defocus caused by refractive errors (shown in the first column) The second and third columns illustrate the performance of a conventional algorithmic approach to prescription correction (as described in Montalto, Garcia-Dorado, Aliaga, Oliveira, Meng, et al. [8].) and our proposed method, respectively, for the same refractive error. For capturing the results in this figure, we used the experimental hardware in Figure 4. Source images are from DIV2K image dataset [40].

**Fig. 7.**
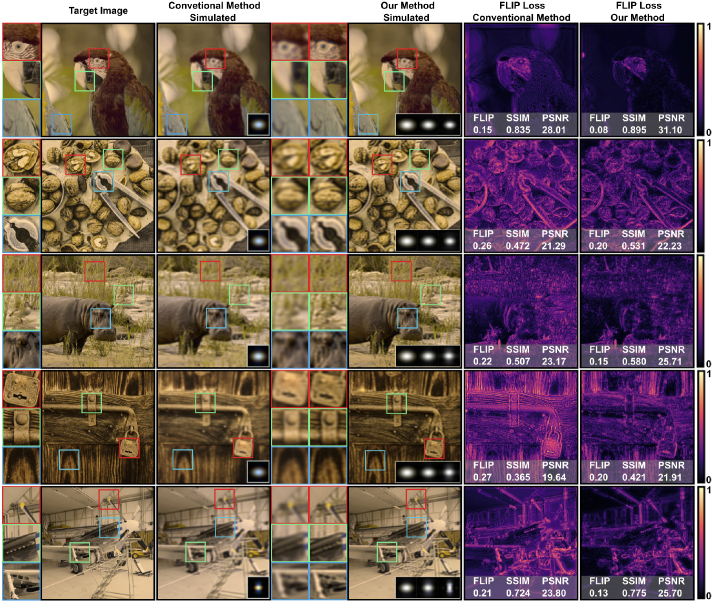
Here we compare outputs from five different refractive vision problems (myopia, hyperopia, hyperopic astigmatism, myopic astigmatism, and myopia with hyperopic astigmatism) for five sample input images. We provide simulated LMS space representations of target image, conventional method output, and our method. FLIP per-pixel difference along with it’s mean value (lower is better), SSIM and PSNR are provided to compare performance of methods. Our method shows better loss numbers for each image quality metrics for each experiment in simulated LMS space. The contrast improvement by using our method against conventional method also can be observed perceptually. Source images are from DIV2K image dataset [40].

In the second part, we evaluated our method with different prescriptions to model various refractive eye problems. Thus, all the images used in this part are evaluated in simulated LMS space. Selected images are aimed to have both high-frequency and low-frequency features. We choose four common cases, myopia, hyperopia, myopic astigmatism, and hyperopic astigmatism, to test our method against the conventional model. Myopia with hyperopic astigmatism represents a complicated refractive eye problem not trivial for prescriptive eyeglasses correction. In each refractive eye problem modeling, +/-1.5 D (Diopters) refractive error is used to model prescriptions. Figure 7 shows our results. We use different image quality measures to compare our method against the conventional method. Our primary image quality metric is FLIP which compares the images using principles of human perception [41]. FLIP allows per-pixel difference loss maps helps to visualize the difference in each pixel against the ground truth image. Therefore, we believe that this metric fits with our work. Although many research on this area has been used SSIM or PSNR loss, FLIP is advantageous as it is adhering human visual system while others are not [42]. In addition to FLIP, we use SSIM and PSNR to compare our method against to conventional method to be stayed relevant with the research community. Figure 7 demonstrates the comparison of our method against the naive method with our perceptually guided color modeling.

**Fig. 8.**
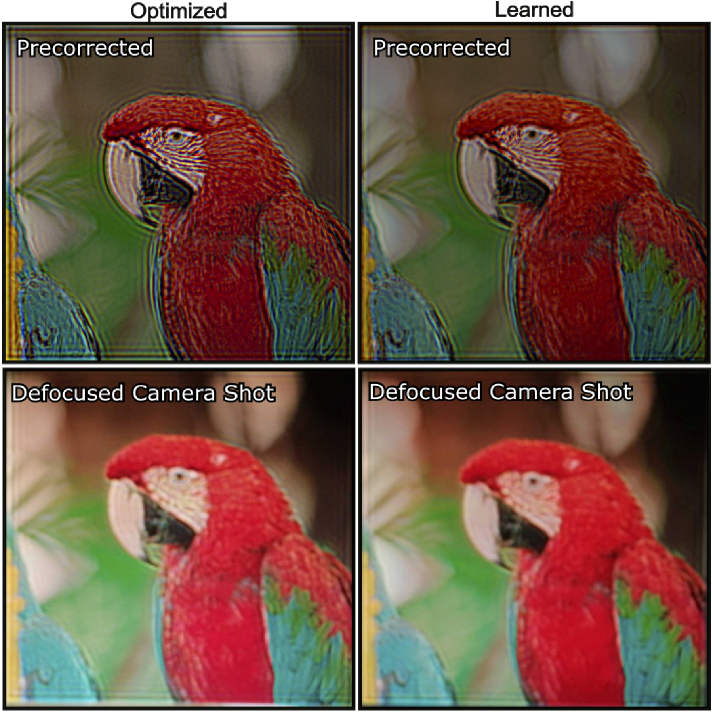
Results from our learned model. We compare our optimization pipeline against our learned model. The top row shows precorrected images reconstructed by our optimizer (Fig. 3) and neural network based learned model. The bottom row shows defocused camera shots for -1.50 myopia by using our defocused camera evaluation method.

Results shows that color opponency based kernel modeling improves the contrast of retinal output image. Selected three areas are mangified to show visibility of improvements in a detailed way. From the per-pixel difference loss maps, we found that our method is better in low-frequency features, while our method also provides improvement in high-frequency parts of images. Overall, it is shown that perceptually guided color based kernel has better contrast compared to conventional method.

## 6. Discussion

To our knowledge, we provide encouraging results improving the conventional method in the literature. However, there are multiple ways to improve our work in the future, we will highlight those in the coming paragraphs.

### Spatially Varying PSF.

Our method does not account for spatially varying natures of PSF in the Human Visual System, which often arrives with computational cost and complexity [43]. We designed our implementation in constant resolution displays instead of varying resolution ones like foveated displays. As an alternative, the deep learning methods can help support spatially varying PSF convolutions in the modeling [44] with lesser computational cost but with demand in data for training. Thus, our method can benefit from these techniques in the future for precision modeling.

### Chromatic Aberrations In A Human Eye.

We use PSF created by the same Zernike coefficients for each wavelength in our forward model. However, optics of Human Visual System contain chromatic aberrations that are wavelength-dependent. As a future work, we can further improve the accuracy of our modeling for a human observer by taking into account the chromatic aberrations in the Human Visual System. In the meantime, curious readers can find greater details regarding chromatic aberrations in work by Cholewiak, Love, Srinivasan, Ng, Banks, et al. [45].

### Image Quality.

Approaches for prescription correction with additive displays are fundamentally limited. This limit stems from the fact that PSF, the non-negative transfer function of an additive display, could support a limited range of frequencies and cause contrast loss. Our work could be made to be complementary to holographic displays [27,28,46], which promise a unique solution for this issue originating from non-negativity in additive displays.

### Foveated Rendering.

**Fig. 9.**
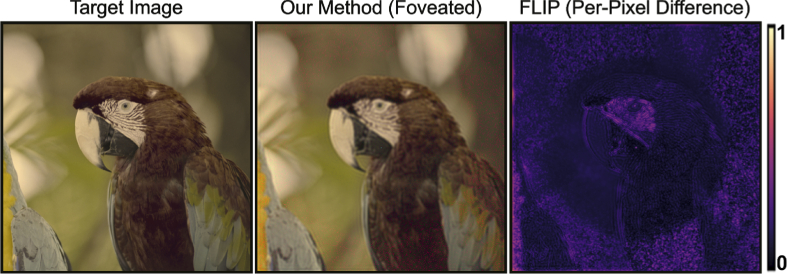
We reconstructed image in our method with addition of foveation. Foveated rendered area is in the center of reconstructed image. FLIP per-pixel difference map highlights the foveation.

Foveated rendering in graphics [47] and displays [48] has garnered interest in the VR and AR research community. We believe that our method can also benefit from this trend by accounting for trends in chromatic and achromatic contrast sensitivity [49–51] in the Human Visual System. Moreover, we could add a rod’s response to cone responses by reformulating the LMS response to improve color difference predictions [52]. We will explore this path in our future work (See Fig. 9 for our early results.)

Moreover, Our method could be potentially integrated with Mandl, Langlotz, Ebner, Mori, Zollmann, Mohr, Kalkofen, et al. [53] to support a broader user base with refractive vision impairments. On the other hand, in our work, we are not also addressing the solution to the color deficiency problem [54], which could also be important for supporting a larger user base.

## 7. Conclusion

Identifying means to help display users with their vision impairments is an essential aspect of graphics systems. As we focus on this critical issue, we present a new rendering approach that provides sharp images when viewed by users with vision impairments without their prescription glasses. Specifically, our rendering approach uniquely merged key insights from HVS. It showed that it could help improve visual experiences and comfort in VR headsets by enhancing color and contrast in the displayed images. The future will likely bring more principled approaches in AR/VR displays (e.g. holographic displays), which could enable future research investigations based on findings from this work.

## Data Availability

All data needed to evaluate the conclusions in the manuscript are provided in the manuscript. Additional data related to this paper may be kindly requested from the author.

## Supplemental document

See Supplement 1 for supporting content.
