# Supplementary material for: ChromaCorrect: prescription correction in virtual reality headsets through perceptual guidance
Source: Biomed Opt Express. 2023 Apr 21;14(5):2166–80. doi: 10.1364/BOE.485776 (PMC10191670; doi:10.1364/BOE.485776)
Supplement: Supplementary file 1 [file boe-14-5-2166-s001.pdf]

Supplemental Document

Biomedical Optics EXPRESS

ChromaCorrect: prescription correction in virtual reality headsets through perceptual guidance: supplement

AHMET H. GÜZEL,[1] JEANNE BEYAZIAN,[2] PRANEETH CHAKRAVARTHULA,[3] AND KAAN AKŞIT[2,*]

[1] *University of Leeds, School of Computing, Leeds, UK*
[2] *University College London, Computer Science Department, London, UK*
[3] *Princeton University, Princeton, USA*
[*] *k.aksit@ucl.ac.uk*

This supplement published with Optica Publishing Group on 21 April 2023 by The Authors under the terms of the Creative Commons Attribution 4.0 License in the format provided by the authors and unedited. Further distribution of this work must maintain attribution to the author(s) and the published article's title, journal citation, and DOI.

Supplement DOI: https://doi.org/10.6084/m9.figshare.22586458

Parent Article DOI: https://doi.org/10.1364/BOE.485776

ChromaCorrect: Prescription Correction in Virtual Reality Headsets through Perceptual Guidance: Supplemental Document

AHMET H. GÜZEL[1], JEANNE BEYAZIAN[2], PRANEETH CHAKRAVARTHULA[3], AND KAAN AKŞIT[2]

[1] *University of Leeds, School of Computing, Leeds, UK*
[2] *University College London, Computer Science Department, London, UK*
[3] *Princenton University, Princeton, USA*
[1] *od20ahg@leeds.ac.uk*
[2] *k.aksit@ucl.ac.uk*
[2] *jeanne.beyazian.21@ucl.ac.uk*
[3] *praneethc@princeton.edu*

1. Contributions

Here, we list contributions from each author in this specific research work:

Ahmet Güzel:

- Coding an entire computational pipeline for color model and optimization.
- Compiling extensive study on evaluation.
- Compiling majority of the figures.
- Writing, review and edits for the entire documentation.

Jeanne Beyazian:

- Compiling a dataset and development and training of machine learning model.
- Helping with the figures.
- Writing sections related to machine learning.

Praneeth Chakravarthula:

- Mentorship (Ahmet Güzel).
- Steering the project towards Virtual Reality applications.
- Review and edits for the entire documentation.

Kaan Akşit:

- Initiation of project idea, this includes blueprints, and early experimentations.
- Literature review and mentorship (Ahmet Güzel and Jeanne Beyazian).
- Writing, review and edits for the entire documentation.

2. Color Opponency

Human retinal cells could be broadly classified into rods and cones. Cone cells are primarily responsible for color perception in Human Visual System (HVS), and the cone density on our retinas peek at our fovea and drops sharply towards larger eccentricities [1]. Cone cells have three subtypes known as Short (S), Medium (M), and Long (L) cells, where each differs in sensitivity to wavelengths of light [2, 3]. These L, M, and S cones reduce wavelengths of incoming light into trichromat values by integrating them over their response functions [4]. According to earlier color opponency studies, HVS relies on comparison of L, M and S cone activation outputs [5]. A widely used modeling method in color opponency is S versus (M+L) for blue-yellow (BY) channel, and L versus M for red-green (RG) channel [6]. Schmidt et al. [7] proposes L versus (M+S) opponency for BY channel and M versus (L+S) opponency for RG channel.

3. Learned Model

Figure 1 shows further results for comparison of the corrected image between our open-box model and the neural network's prediction after over 800 epochs of training.

4. Futher Evaluation

Figure 2 shows further results for different myopia starting from -2.0 dioptres to -4.5 dioptres. We show that our method's reconstructed images have better constrast than conventional method's reconstruced images for each case. Images are simulated in LMS space.

Figure 3 shows further results for different images from DIV2K image dataset [8]. In each refractive eye problem modelling, +/-1.5D refractive error is used to model prescriptions.

References

1. J. B. Jonas, U. Schneider, and G. O. Naumann, "Count and density of human retinal photoreceptors," Graefe's Arch. for Clin. Exp. Ophthalmol. **230**, 505–510 (1992).
2. A. Stockman and L. T. Sharpe, "The spectral sensitivities of the middle- and long-wavelength-sensitive cones derived from measurements in observers of known genotype," Vis. Res. **40**, 1711–1737 (2000).
3. A. Stockman, L. T. Sharpe, and C. Fach, "The spectral sensitivity of the human short-wavelength sensitive cones derived from thresholds and color matches," Vis. Res. **39**, 2901–2927 (1999).
4. S. Wuerger, "Colour perception," in *London Imaging Meeting,* vol. 2022 (Society for Imaging Science and Technology, 2022), pp. YZ–MK.
5. L. M. Hurvich and D. Jameson, "An opponent-process theory of color vision." Psychol. review **64, Part 1 6**, 384–404 (1957).
6. D. H. B. Andrew Stockman, "Color vision mechanisms," in *Vision and Vision Optics 3rd,* M. Bass, ed. (McGraw-Hill, New York, 2009), chap. 11, pp. 1–104.
7. B. P. Schmidt, M. Neitz, and J. Neitz, "Neurobiological hypothesis of color appearance and hue perception." J. Opt. Soc. Am. A, Opt. image science, vision **31 4**, A195–207 (2014).
8. E. Agustsson and R. Timofte, "Ntire 2017 challenge on single image super-resolution: Dataset and study," in *The IEEE Conference on Computer Vision and Pattern Recognition (CVPR) Workshops,* (2017).

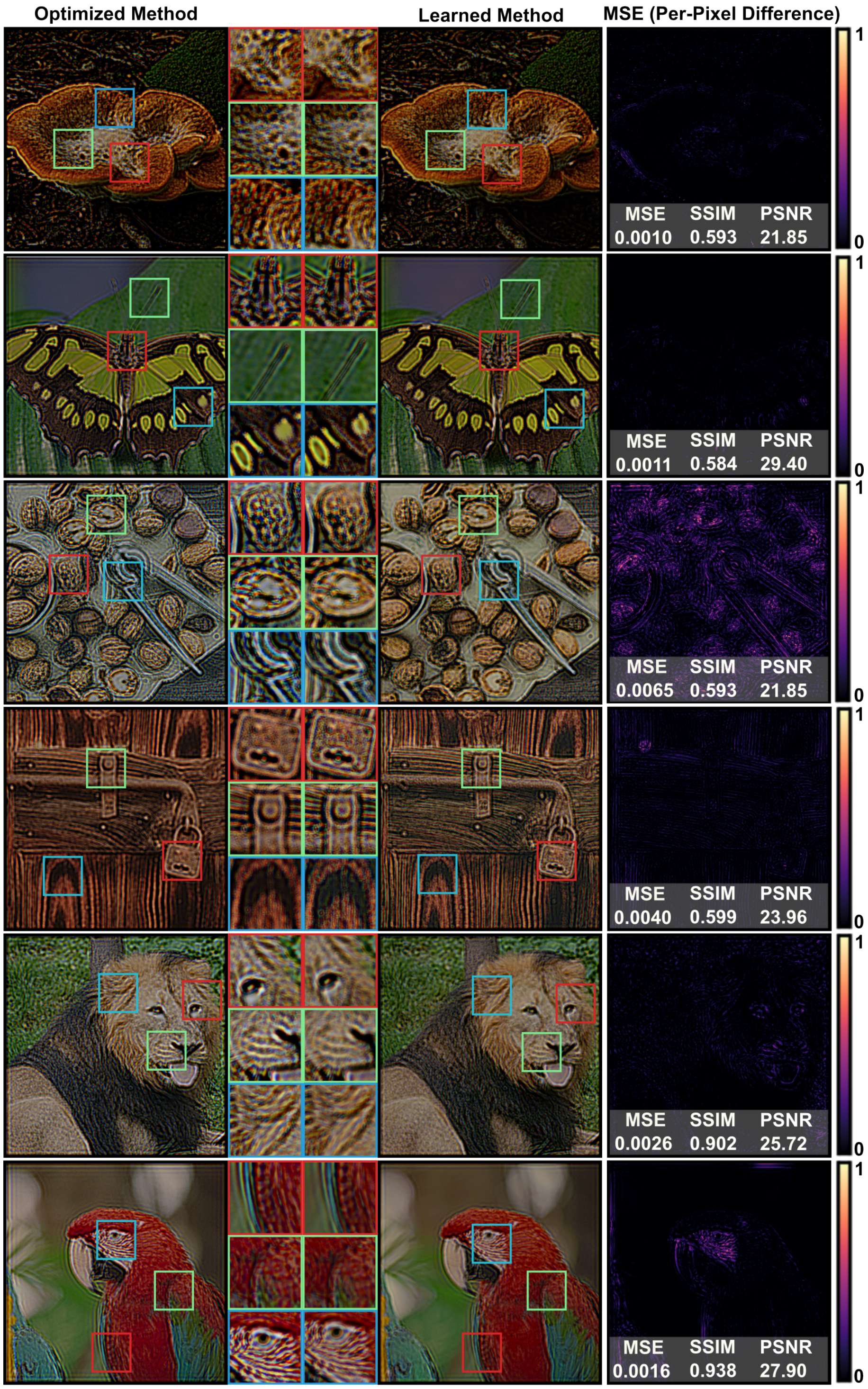

Fig. 1. Additional images for learned method. Images are selected from DIV2K image dataset [8].

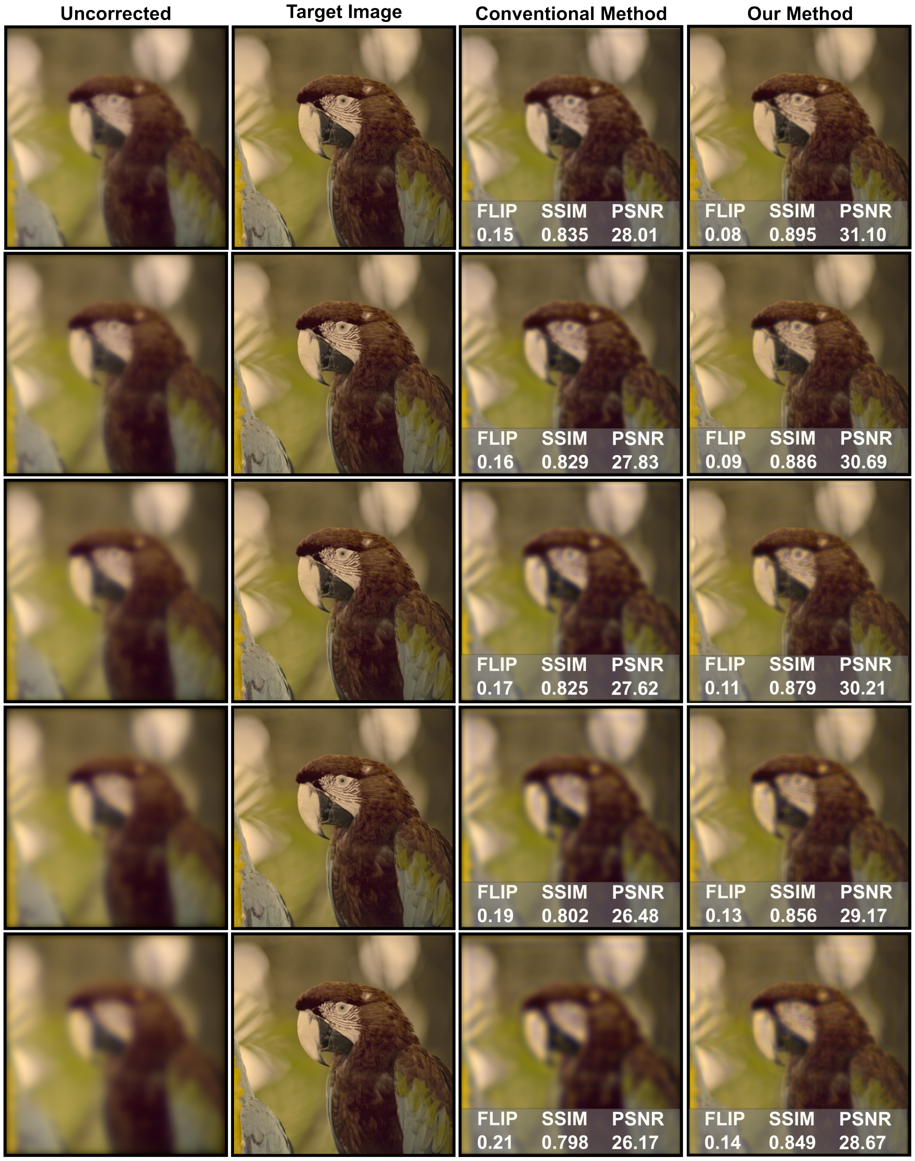

Fig. 2. Comparison of convetional method and our approach with different myopia cases. Images are simulated in LMS space.

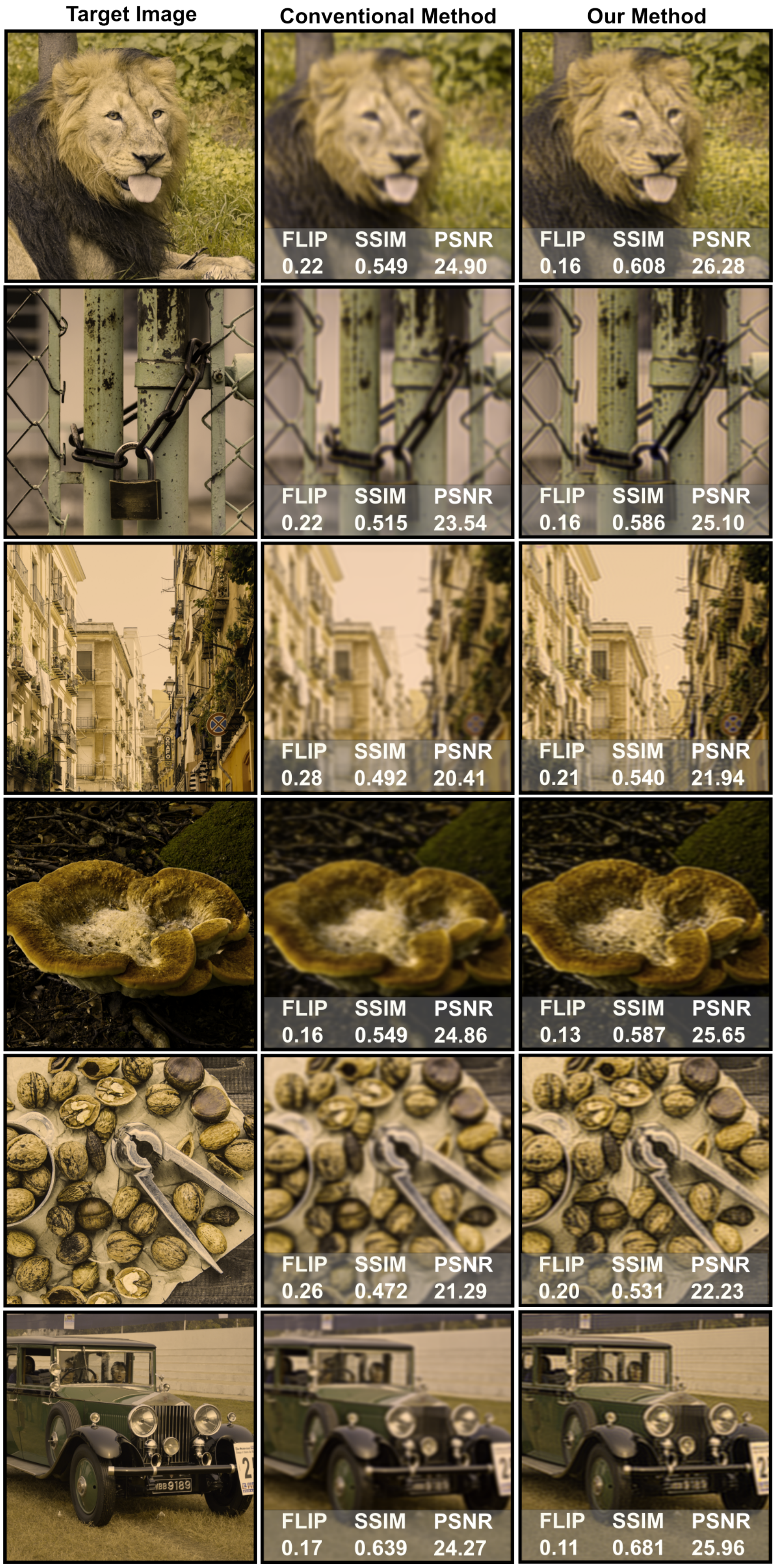

Fig. 3. Additional images for the evaluation section. Images are simulated in LMS space.
